# Computer vision-based support system for B-line detection in lung ultrasound

**DOI:** 10.3389/fdgth.2026.1917310

**Published:** 2026-08-12

**Authors:** Julia López-Canay, Manuel Casal-Guisande, Cristina Ramos-Hernández, Maribel Botana-Rial, Alberto Fernández-Villar

**Affiliations:** 1 NeumoVigo i+I Research Group, Galicia Sur Health Research Institute (IIS Galicia Sur), SERGAS-UVIGO, Vigo, Spain; 2 Department of Design in Engineering, University of Vigo, Vigo, Spain; 3 Centro de Investigación Biomédica en Red, CIBERES ISCIII, Madrid, Spain; 4 Pulmonary Department, Hospital Álvaro Cunqueiro, Vigo, Spain; 5 Department of Functional Biology and Health Sciences, University of Vigo, Vigo, Spain

**Keywords:** B-lines, computer vision, lung ultrasound, object detection, YOLO

## Abstract

**Context and objectives:**

Lung ultrasound (LUS) is a safe and cost-effective diagnostic tool. B-lines are fundamental ultrasound (US) artifacts for LUS-based diagnosis of various pulmonary conditions, especially for the evaluation of the lung parenchyma. However, the challenges in their identification and interpretation, combined with a shortage of experts and training programs, restrict the use of this tool in routine clinical practice.

**Methods:**

To overcome these limitations, this study presents a computer vision (CV)-based system for automatic B-line detection. The proposed system integrates preprocessing and feature engineering stages with an object detection module. First, LUS images are normalized to ensure interoperability across different US devices and settings. Subsequently, the Radon and inverse Radon transforms are applied to generate a mask that highlights hyperechoic vertical structures, which is then fused with the preprocessed LUS image. Finally, the resulting image serves as input for a convolutional neural network (CNN) based on the You Only Look Once (YOLO) architecture, enabling the automatic localization of B-lines.

**Results:**

The results obtained on the test set demonstrate satisfactory performance, achieving a precision of 89.13%, a recall of 80.39%, and an average precision (AP) of 0.82 at an Intersection over Union (IoU) of 0.5.

**Conclusions:**

A clinical decision support tool is proposed, aimed at improving efficiency and consistency in LUS interpretation, as well as facilitating its integration into clinical practice. Although the system is still in its conceptual stage, these findings lay the groundwork for future clinical validation processes directed toward its future implementation.

## 1 Introduction

Ultrasound (US) is widely used in medical practice due to its diagnostic utility across diverse pathologies and its role in guiding clinical interventions (1). Lung ultrasound (LUS) offers multiple advantages over other imaging technologies, such as computed tomography (CT) and chest radiography, including its safety, repeatability, flexibility, and cost-effectiveness. These features make it well-suited for bedside examinations using portable devices (2, 3).

LUS diagnosis of pulmonary conditions depends on the identification and interpretation of US artifacts. Far from being a limitation, these artifacts are fundamental to clinical assessment, particularly when evaluating the lung parenchyma (4), as they arise from acoustic impedance differences between the pleura (tissue) and the lung (air/fluid) (5). Historically, LUS was confined to Emergency and Critical Care departments, while its use in Pulmonology was limited to interventional units (2, 3, 6). However, following the COVID-19 pandemic, its adoption has expanded rapidly across general clinical Pulmonology. By enabling bedside evaluation and minimizing intra-hospital patient transfers, LUS reduces exposure and transmission risks (6). Furthermore, its application in Primary Care helps guide clinical decision-making, reducing the need for complementary studies or secondary care referrals (7).

However, the widespread adoption of LUS in Pulmonology and Primary Care faces significant challenges. For instance, Ramos-Hernández et al. (8) reported that while 91.3% of the surveyed pulmonologists use LUS to identify and evaluate pleural effusions, its application in other indications remains limited. This limitation is primarily attributed to a lack of standardized training, with only 5.7% of professionals identifying as experts (8). Similarly, in Primary Care, recent surveys reveal that although 47% of practitioners have received LUS training, only 18% use it regularly (9). Beyond training gaps, image interpretation presents a major barrier to clinical integration. Image quality and visualization depend on factors such as operator experience, patient anatomy, and device settings. These variables directly affect the identification of lung artifacts, such as B-lines (10), making their assessment a complex task subject to significant interobserver variability (11).

Currently, artificial intelligence (AI)-based tools are reshaping clinical practice by offering advanced decision-making solutions that optimize patient care and administration of healthcare resources (12–16). Computer vision (CV) is a branch of AI focused on the processing, analysis, and interpretation of images and videos, aiming to detect, identify, and localize patterns, objects, or scenes, partially emulating human visual capabilities (17, 18). In clinical settings, CV techniques enhance diagnostic imaging (19), facilitate the monitoring and follow-up of pathologies and surgical procedures, reduce response times (20), and strengthen the efficiency of traditional diagnostic tools (13, 21). Furthermore, integrating CV into simulators and training programs further underscores its importance in advancing clinical practice (20).

In the context of LUS, the development of CV-based tools to identify lung artifacts remains an emerging field. The systematic review conducted by López-Canay et al. (22) highlights significant heterogeneity across existing methodologies, ranging from segmentation strategies to classification models paired with saliency maps. However, segmentation strategies (23–32) often require heavy, complex architectures that impede real-time performance. Meanwhile, while saliency maps offer explainability for classification models (33–42), they do not explicitly solve object localization tasks. To address these limitations, object detection models have gained relevance, as they enable the simultaneous identification and localization of lung artifacts and anatomical structures in LUS images.

Xing et al. (43) developed a system using region-based convolutional neural networks (R-CNN) (44). Conversely, Bottino et al. (45) and Tripathi et al. (46) employed single-stage You Only Look Once (YOLO) networks (47), while Joseph et al. (48) applied KeyNet (49) for keypoint detection. Among these works, only three studies (45, 46, 48) target B-line identification, although they present notable limitations. Methodological deficiencies include a lack of validation on independent datasets and the omission of standard metrics, such as Intersection over Union (IoU) or average precision (AP). Specifically, only Joseph et al. (48) reported AP, achieving a moderate performance score of 0.65.

To address these gaps, this study proposes the design, development, and implementation of a CV-based object detection system for the automated identification and localization of B-lines in LUS images. The proposed framework aims to support LUS interpretation and assist in diagnosing respiratory pathologies by detecting, localizing, and quantifying B-lines. Ultimately, this approach seeks to reduce operator dependence and promote the clinical adoption of LUS in settings where it remains underutilized, such as Pulmonology and Primary Care.

This paper is organized into five sections. Section 1 defines the general framework of the proposed CV system. Section 2 presents the materials and methods, with Section 2.1 presenting the database, Section 2.2 outlining the conceptual design, and Section 2.3 describing the system's implementation as a software artifact. Next, Section 3 presents the results obtained on a reserved test set and demonstrates its operation through proof-of-concept case study. Section 4 provides a discussion of the work performed. Finally, Section 5 presents the final conclusions.

## 2 Materials and methods

### 2.1 Database

To develop the CV system for B-line detection in LUS, the publicly available LUS-BALD dataset (50) was utilized. This dataset comprises 331 labeled images containing B-lines, derived from LUS scans performed on 152 patients (113 men and 39 women; mean age: 40.91 ± 17.03 years) recruited for a clinical study at the Mulago and Kiruddu National Referral Hospitals (Uganda). These patients presented with suspected COVID-19 or other pulmonary pathologies, including various types of pneumonia. Scans were conducted by three expert radiologists, each with over 10 years of LUS experience, following a 12-zone protocol: six zones per hemithorax (upper/lower anterior, lateral, and posterior). Both longitudinal and transversal scans were performed in each zone, acquiring 24 frames per patient. The equipment used included two commercial convex probes: the Clarius C3 (Clarius Mobile Corp., Burnaby, BC, Canada) and the Philips Lumify C5-2 (Philips Ultrasound, Bothell, WA, USA).

The dataset was partitioned into training, validation, and test sets. The characteristics of each subset, including the number of images, total number of B-lines and the average number of B-lines per image, are summarized in Table 1.

**Table 1:** Dataset characteristics across training, validation, and test sets.

Characteristic	Training set	Validation set	Test set
Number of images	264	33	34
Number of B-lines	373	49	51
Average B-lines per image	1.41	1.48	1.50

### 2.2 Conceptual design

**Figure 1:**
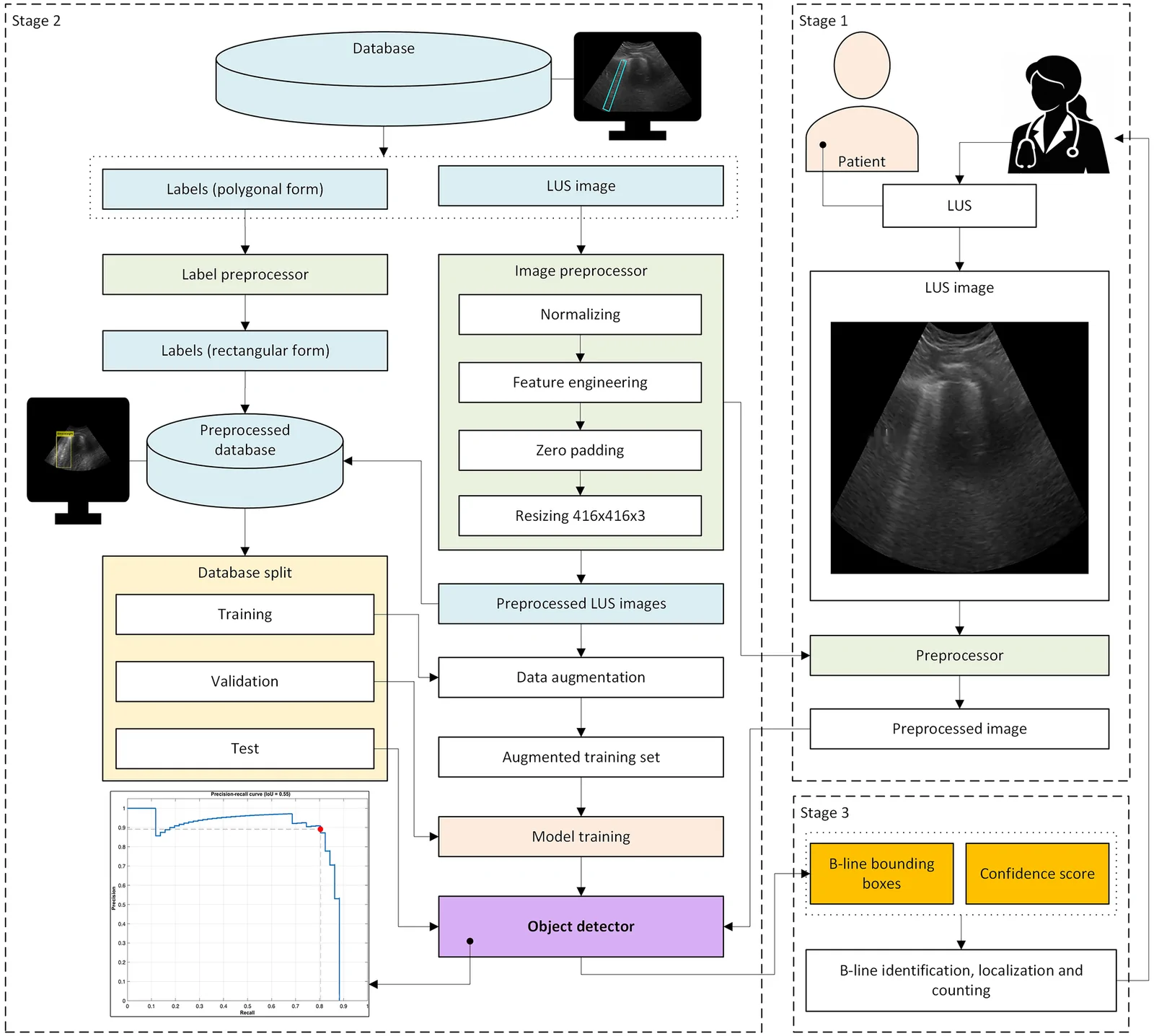
Flowchart of the proposed CV system.

Figure 1 illustrates the flowchart of the CV system for supporting LUS interpretation through the detection and localization of B-lines. A detailed description of the different stages is presented below.

#### 2.2.1 Stage 1 - data collection

The proposed workflow begins with clinical data collection and preprocessing. Specifically, this requires loading a frame from a prior LUS examination into the system to evaluate it for the presence of B-lines.

#### 2.2.2 Stage 2 - data processing

Following data collection, the LUS frame is processed by the CV system. First, the image undergoes a preprocessing and feature engineering stage designed to enhance the visibility of hyperechoic vertical structures. This stage comprises the following steps:

- Image preprocessing: Initially, normalization is carried out to adjust the pixel intensities to a [0, 255] range. Subsequently, distance gain attenuation (DGA) is applied to homogenize brightness along the vertical axis of the image.
- Feature engineering: This step aims to highlight the areas of vertical hyperechogenicity in the image, facilitating the identification of B-lines. This process consists of generating a mask through the application of directional filtering based on the Radon Transform (51). As a result, a composite image is obtained by fusing the preprocessed image with the mask.

The resulting composite image is then processed by an object detection system based on the YOLO architecture (47). This architecture enables simultaneous identification and localization of objects, thereby allowing for the precise localization of the B-lines present in the LUS image.

#### 2.2.3 Stage 3 - LUS interpretation and decision support

Once the system processes the LUS image, bounding boxes are generated to locate the B-lines within the image, along with their associated confidence scores. Additionally, the system provides an automatic count of the detected B-lines and indicates whether the finding is considered pathological.

These results provide clinicians with a support tool for LUS interpretation, enabling precise assessment based on the presence, absence, or density of B-lines. In this way, the system supplies objective and quantifiable information, which is essential for optimizing clinical decision-making and patient management.

### 2.3 System implementation

The system was developed in the MATLAB^©^ environment (version R2025b, Natick, MA, USA). The Image Processing Toolbox (52) was utilized for image preprocessing and feature engineering. Label management and transformation were performed using the Image Labeler app (53), supported by the Computer Vision Toolbox (54) and Deep Learning Toolbox (55). These toolboxes were also employed to train the CV system. Finally, the user interface was designed and implemented using App Designer (56). The hardware used for the system's implementation consists of an Apple M2 Pro processor (Apple Inc., CA, USA) featuring a 16-core integrated GPU and 16 GB of RAM.

**Figure 2:**
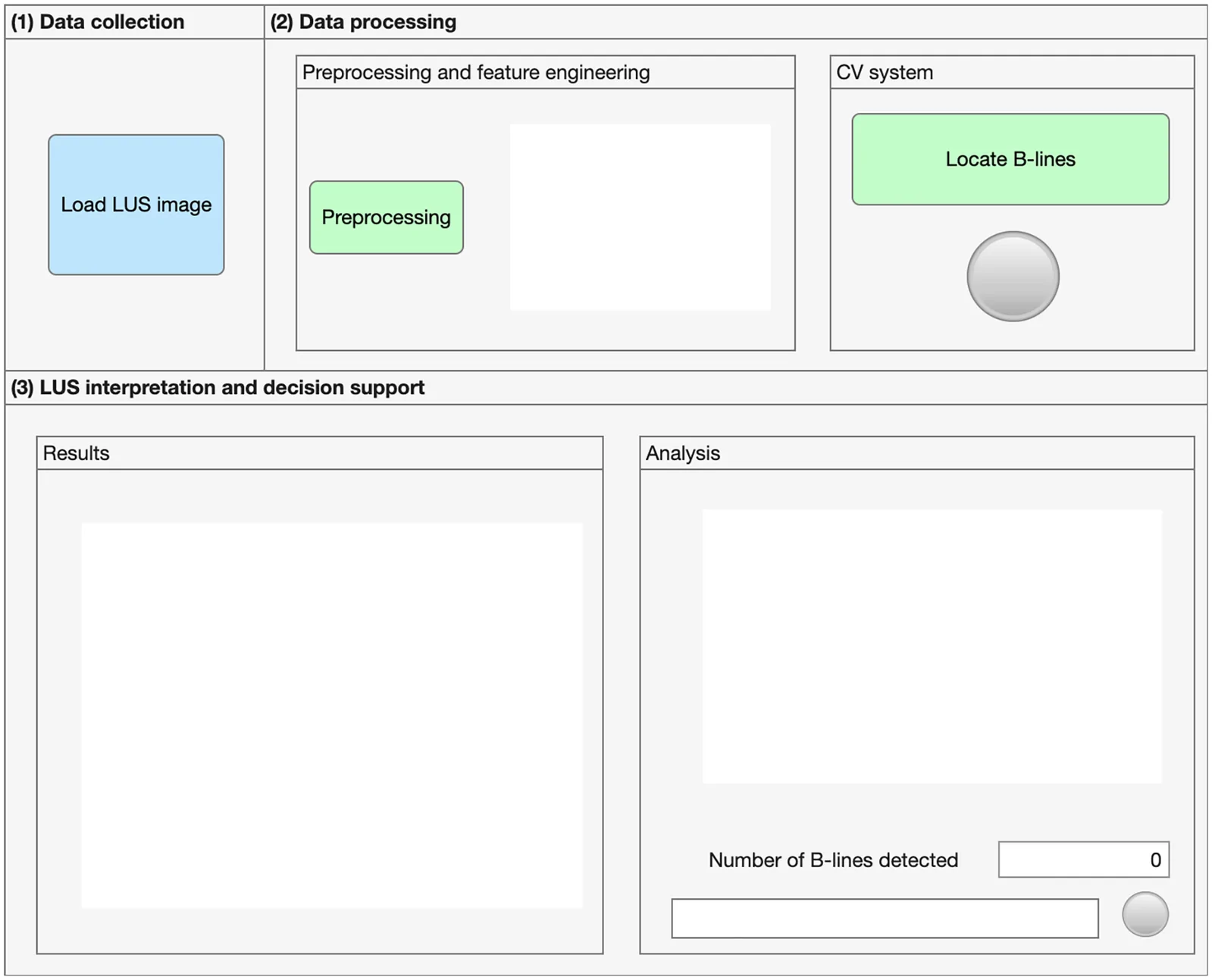
System user interface.

The workflow initiates with the user loading the target LUS image into the CV system. This action is executed via the “Load image” button within section (1) of the graphical user interface (Figure 2).

#### 2.3.1 Stage 2 - data processing

After loading the LUS image, the system initiates a preprocessing flow structured into three fundamental phases: preprocessing, feature engineering, and geometric adjustment. This workflow ensures the optimization and standardization of the input image in accordance with the requirements of the YOLOX network (57).

**Figure 3:**
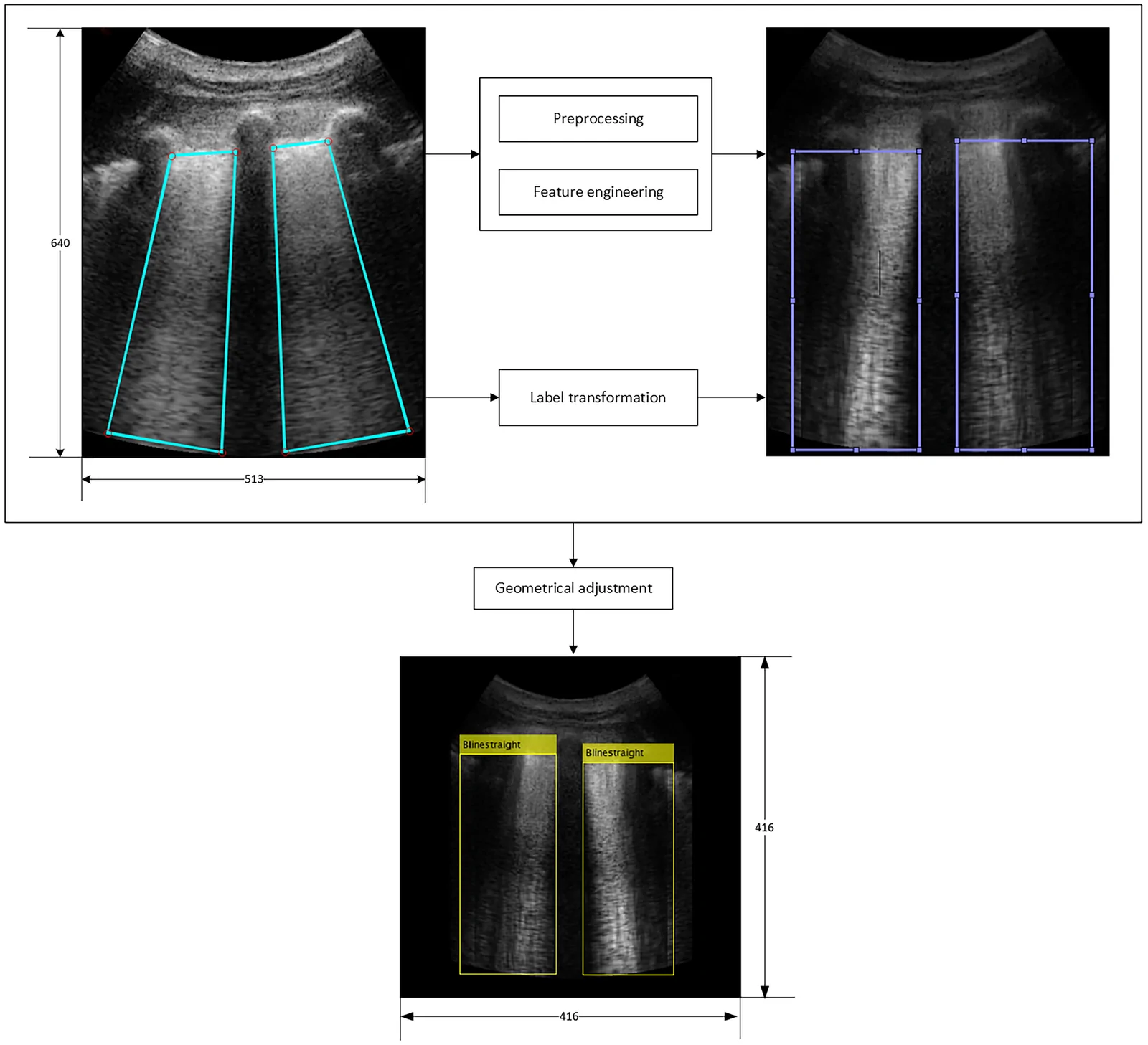
Image preprocessing pipeline and label adjustment.

To ensure the system's functionality, a prior training phase is required. During this stage, the labels associated with the images undergo denormalization and coordinate transformation (Figure 3) to rigorously adapt them to the technical specifications and input format required by the network.

Once model training is complete and the patient's image has been processed, the CV system executes the inference phase. As a result, it generates bounding boxes that identify the location of the B-lines within the image. Each detection includes a confidence score that quantifies the probability that the indicated region corresponds to a B-line.

##### 2.3.1.1 Preprocessing, feature engineering, and geometric adjustment

To ensure system performance remains independent of the US equipment and acquisition parameters used during the LUS scan, pixel intensity normalization is performed. This process guarantees a consistent visual appearance and reduces inter-case variability by standardizing the dynamic range of the images.

First, the RGB image is converted to grayscale. Subsequently, Min-Max normalization is applied to scale gray levels to the [0, 255] range using Equation 1, where *I* represents the original image and *Inorm* denotes the normalized image. Through this linear transformation, the minimum intensity level of the scan is redefined as 0 and the maximum as 255, proportionally redistributing the intermediate values. This technical adjustment ensures a standarized input for the CV model, mitigating biases derived from the initial lighting or contrast conditions of the examination.

$$I_{norm}=\frac{I-min(I)}{max(I)-min(I)}\cdot 255$$ (1)

B-lines correspond to hyperechoic vertical structures that extend toward the bottom of the image. Due to the natural attenuation of the US beam, as well as the attenuation resulting from its interaction with various tissues, signal intensity decreases with depth. To compensate for this phenomenon and ensure that the system remains independent of the gain parameters used during image acquisition, a DGA process is performed.

To achieve this, a depth vector *d* (from 0 to 16 cm) is defined so that each row of the image corresponds to a physical depth. Subsequently, an exponential gain curve is generated according to Equation 2, using an adjustment coefficient *a* = 0.05. In this manner, each row of pixels in the image is multiplied by the gain factor corresponding to its depth, highlighting the complete trajectory of the B-lines.

$$I_{DGA}=I_{norm}\cdot \left(1-\frac{e^{-a\cdot d}}{max(e^{-a\cdot d})}\right)$$ (2)

Subsequently, a feature engineering process is performed to facilitate the localization of the B-lines present in the image. This process highlights structures of interest corresponding to the hyperechoic vertical lines within the image. To achieve this, the Radon Transform (51) and its inverse are used in combination. This technique filters the original image, emphasizing specific structures based on their geometric orientation.

First, the Radon Transform of the image is calculated, projecting the image's intensity along radial lines oriented at specific angles. Although B-lines possess an essentially vertical morphology, the use of convex probes induces a fan-like array, resulting in a progressive angular inclination relative to the central axis of the frame. To compensate for this geometric divergence, the transform is calculated selectively for the angular ranges of [0, 20] and [160, 180]. The result of this operation is an optimized sinogram: a representation in the Radon domain where each column stores the intensity projection for the angles of interest, facilitating the discrimination of pathological lines from background noise.

Next, the inverse Radon Transform is applied to the generated sinogram. This process reconstructs the image in the spatial domain using exclusively the information from the filtered angles, producing a semantic mask that highlights the hyperechoic vertical structures while suppressing background noise.

Subsequently, the generated mask is integrated with the original image using an alpha blending technique according to Equation 3. In this operation, the parameter *α* determines the weighting of the mask over the final image. For this implementation, a value of *α*=0.7 was selected, allowing the Radon mask to contribute 70% of the visual weight, while the preprocessed image retains the remaining 30%.

$$I_{\;final}=I_{norm}\cdot (1-\alpha )+I_{mask}\cdot \alpha$$ (3)

Finally, to meet the neural network's input requirements and avoid introducing morphological distortions, a zero-padding step is performed prior to image resizing. This process adds a black border around the image, using its largest dimension as a reference to produce a square image. This prevents the image from being stretched or distorted during the subsequent resizing step, where it is scaled to a resolution of 416 × 416 × 3 pixels, the standard input size for YOLO architectures (47), thereby ensuring that spatial relationships are preserved.

##### 2.3.1.2 Definition of the CV system

Once the enhanced image is obtained, it serves as input for the CV system based on the YOLO architecture (47). The model processes the image in a single stage, outputting bounding boxes that precisely locate the B-lines within it. Each detection includes an associated confidence score, which represents the probability that the bounded region corresponds to a B-line.

To achieve this, the system first undergoes supervised training using the training and validation image sets alongside their respective reference labels.

The original labels from the database are presented in a trapezoidal format, composed of normalized *x* and *y* coordinates for each vertex forming the trapezoid (Figure 3). Given that YOLO architectures (47) require a specific input format, a transformation process was performed on the original labels to convert them into a 1 × 4 vector following the [*x*, *y*, *w*, *h*] standard, where:

- *x* and *y*: Correspond to the coordinates of the top-left corner of the bounding box.
- *w* (width): Specifies the width of the rectangle along the horizontal axis.
- *h* (height): Defines the height of the rectangle along the vertical axis.

To perform this transformation, the original labels are first denormalized by multiplying the *x* and *y* components of each trapezoid vertex by the actual width and height of the image. Once these spatial positions are calculated, the extreme values on both axes are identified. The minimum coordinate values define the top-left corner of the box (*x*_min_, *y*_min_), while the maximum values determine the object's boundary. The width and height are then calculated by taking the difference between these extreme values, yielding a label format fully compatible with the standard training pipeline for YOLO networks (47).

Finally, the bounding box coordinates are recalibrated to account for the zero-padding and resizing operations applied to the input image. This adjustment is critical to ensure that the labels remain perfectly aligned with the B-lines present in the images following the geometric transformation of the data.

Once the labels have been processed, the dataset is divided into three independent sets: training (80%, 264 images), validation (10%, 33 images), and testing (10%, 34 images). To mitigate the limitations imposed by the sample size and prevent overfitting, a data augmentation strategy was implemented exclusively on the training set. This strategy applies random transformations to the images to increase sample variability. Specifically, random horizontal reflections were chosen, applied synchronously to both the images and their respective bounding boxes to maintain spatial coherence.

Giving the small dataset size, a transfer learning strategy was implemented, which involves using networks previously trained on larger datasets, thereby avoiding random weight initialization. In this case, the YOLOX object detector (57) was selected. Unlike previous YOLO versions, this architecture allows for single-stage object detection without the need for anchor boxes, guaranteeing real-time system implementation. This anchor-free approach simplifies the training process by predicting object locations directly from their centers rather than adjusting predefined dimensions. Furthermore, YOLOX (57) offers flexible input dimension scaling, facilitating model adaptation to diverse imaging hardware in clinical environments.

The YOLOX network (57) is structured into three main components:

- Backbone: Acts as the core for feature extraction, generating hierarchical feature maps from the input images. It consists of a convolutional neural network (CNN) pre-trained on the COCO dataset (58) based on the CSP-DarkNet-53 architecture (59).
- Neck: Employs a Feature Pyramid Network (FPN) to connect the backbone with the detector head. Its function is to aggregate information from the different hierarchical levels, generating feature maps at three distinct scales (1024, 512, and 256 channels), which facilitates the detection of objects of various sizes.
- Head: Processes the aggregated features to obtain three distinct outputs:
  - Classification score: Determines the object's class. In this case, a single class is utilized (B-lines), so all detected objects belong to the same class.
  - Regression score: Defines the geometry and location of the bounding boxes corresponding to the identified objects.
  - Objectness or confidence score: Combines the IoU index and the class probability. It quantifies both the model's confidence that the box contains the object of interest (B-lines) and the precision of its localization.

Given the morphological complexity of LUS images, the large-scale variant of the network (Large-COCO) was selected. Table 2 details the specific hyperparameter configuration used during model training.

**Table 2:** Training parameters of the CV system.

Hyperparameter	Value	Comment
Optimizer	Adam	–
Mini-batch size	16	–
Maximun number of epochs	100	–
Learning rate	0.0001	–
Validation frequency	20	The maximum number of consecutive iterations allowed without improvement in validation error before training is interrupted.
Output network	Best validation error	Enables the selection of network parameters corresponding to the iteration that achieved the lowest validation error.

#### 2.3.2 Stage 3 - LUS interpretation and decision support

Once the image has been processed, the CV system outputs the bounding boxes for all detected B-lines alongside their corresponding confidence scores. To enhance model explainability, D-RISE saliency maps (60) are integrated. Furthermore, the system evaluates the results by quantifying the number of B-lines and suggesting potential diagnoses based on the observed LUS findings. Altogether, this information constitutes a valuable clinical decision-support tool that assists clinicians in interpreting LUS scans and facilitates the precise identification of these artifacts.

## 3 Results

**Figure 4:**
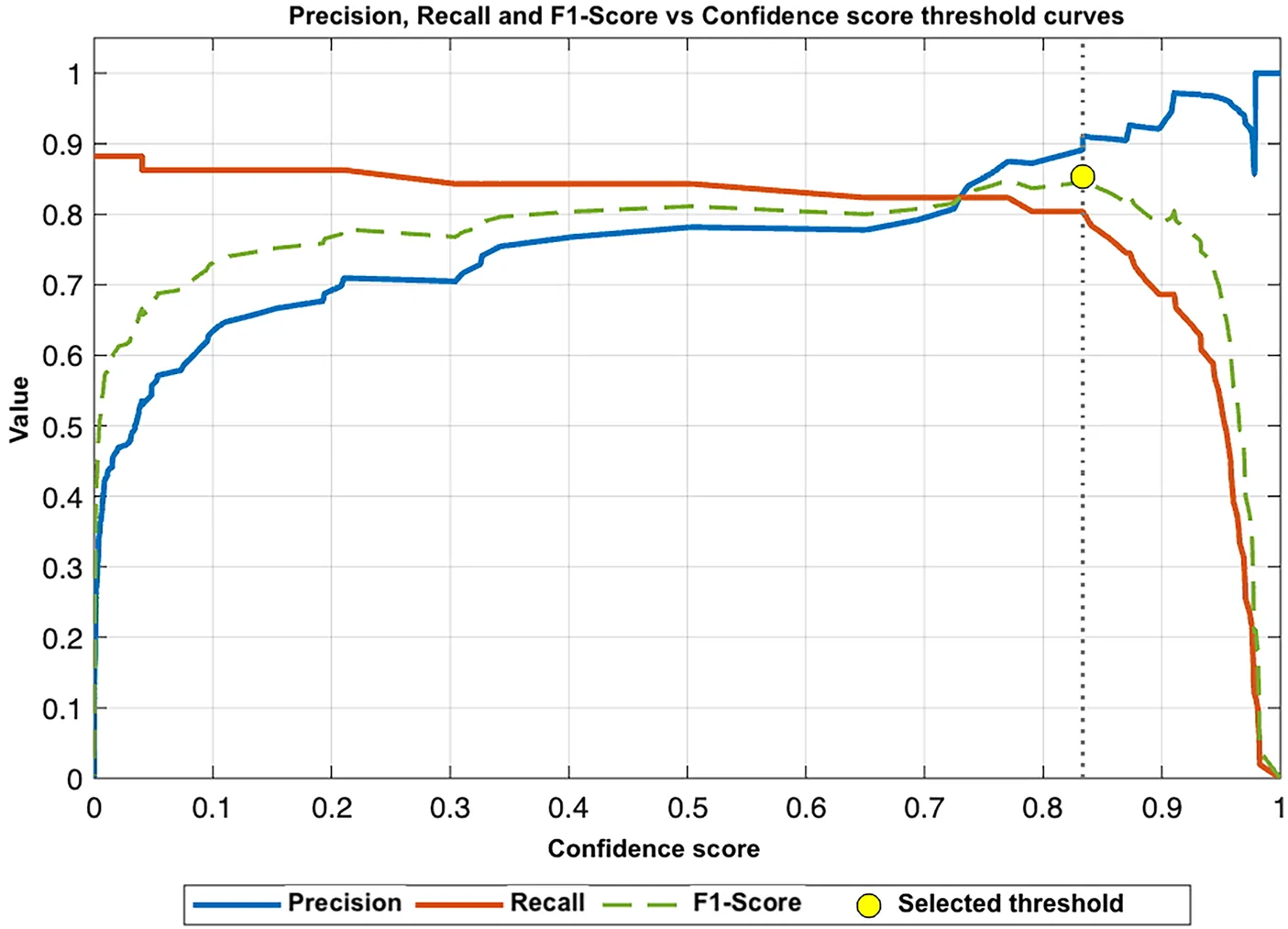
Precision (blue), recall (orange), and F1-score (green) curves as a function of the confidence score. The yellow marker indicates the confidence threshold corresponding to the maximum F1-score.

The performance of the CV system was evaluated on an independent test set to assess its generalization capability. Multiple confidence-score thresholds were evaluated, and precision, recall, and F1-score were calculated at each threshold. The threshold that produced the highest F1-score was selected as the optimal operating cutoff. Based on this analysis, a confidence threshold of 0.83 was selected, yielding a precision of 89.13%, a recall of 80.39% and an F1-score of 84.54% (Figure 4).

The IoU coefficient is a fundamental metric for evaluating object detection models. It quantifies spatial localization accuracy by calculating the ratio of the intersection area between the predicted bounding box and the ground truth label to the total area of their union. This coefficient varies within the range of [0, 1]. Under this criterion, if the IoU equals or exceeds a pre-established threshold, the detection is classified as a true positive, confirming that the predicted bounding box aligns satisfactorily with the ground truth; otherwise, the prediction is considered a false positive. In accordance with the stablished literature, an IoU threshold of 0.5 was selected for model evaluation (19, 48).

**Figure 5:**
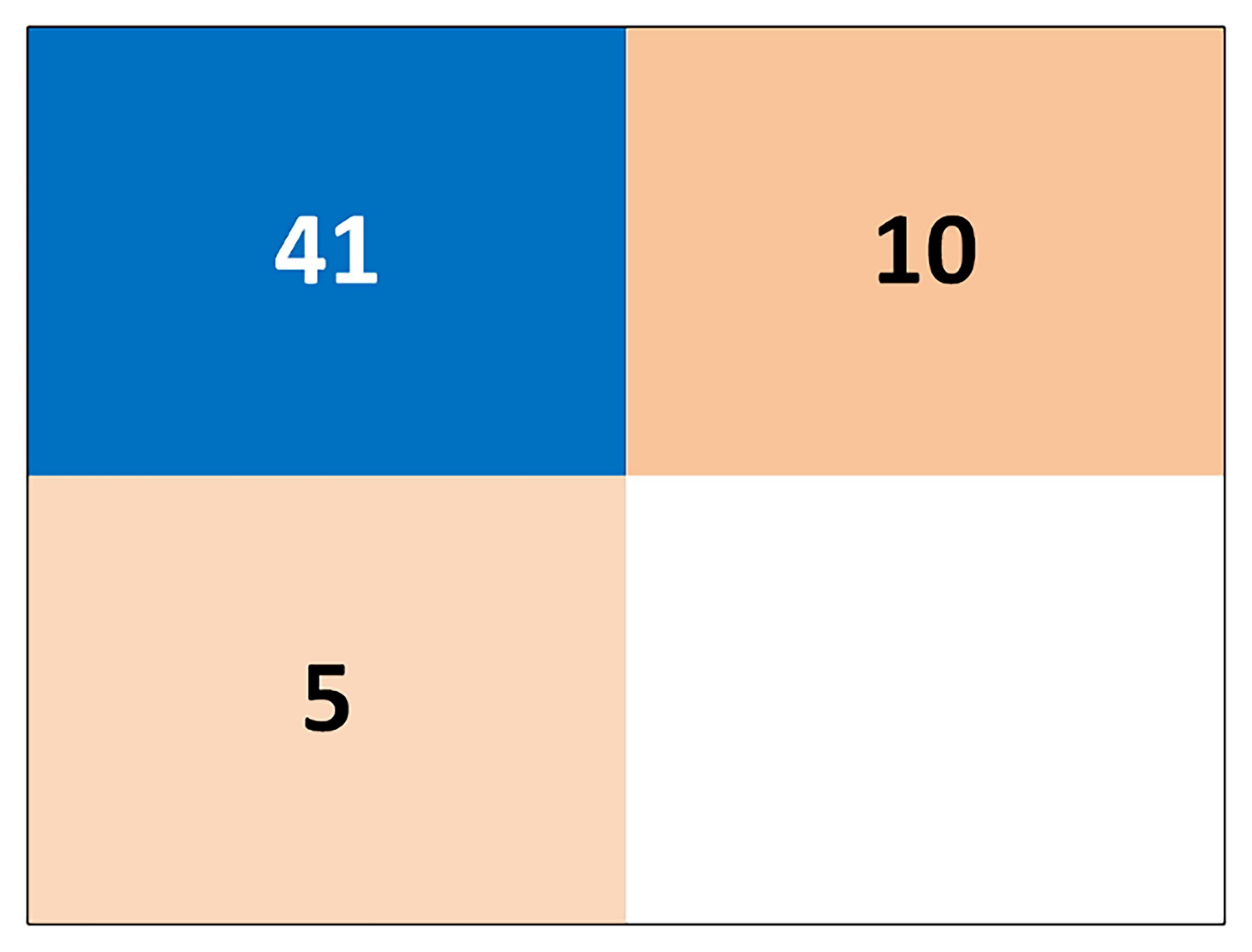
Confusion matrix (IoU = 0.5).

**Figure 6:**
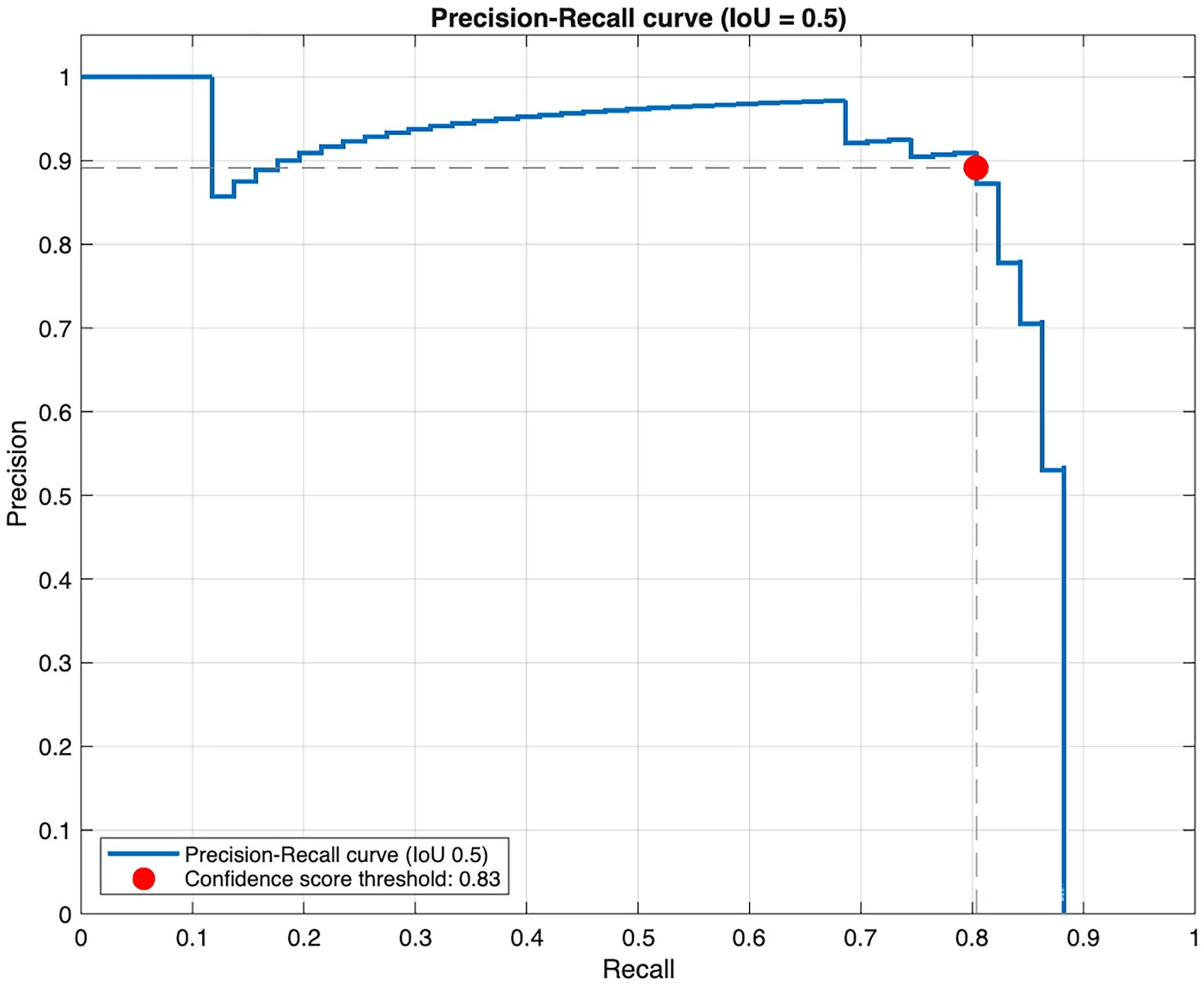
Precision-recall curve (IoU = 0.5). The red point indicates the system's operating point at the selected confidence threshold of 0.83.

Combining the optimal confidence threshold and an IoU threshold of 0.5 yielded the confusion matrix shown in Figure 5. Out of the 51 B-lines present in the test set, the model correctly detected 41, missed 10, and generated 5 false positive detections. Figure 6 presents the precision-recall curve at an IoU threshold of 0.5, with the system's operating point indicated in red.

To enhance model explainability, saliency maps were calculated. Given that neural network-based models are inherently opaque, these techniques are commonly used to facilitate their implementation in clinical practice. These saliency maps identify the image regions that contributed most significantly to the model's predictions.

Specifically, the D-RISE method (60) was selected, as it was designed for generating visual explanations in object detection models based on YOLO architectures (47). By highlighting the key salient regions driving B-line identification, D-RISE improves the interpretability of the CV system, offering clinicians transparent diagnostic rationale.

### 3.1 Case study

Below, the operation of the developed CV system is illustrated using a practical case. It should be noted that the data used in this example were excluded from the model's training set. This scenario serves as a proof of concept and, therefore, does not constitute a formal validation of the system.

**Figure 7:**
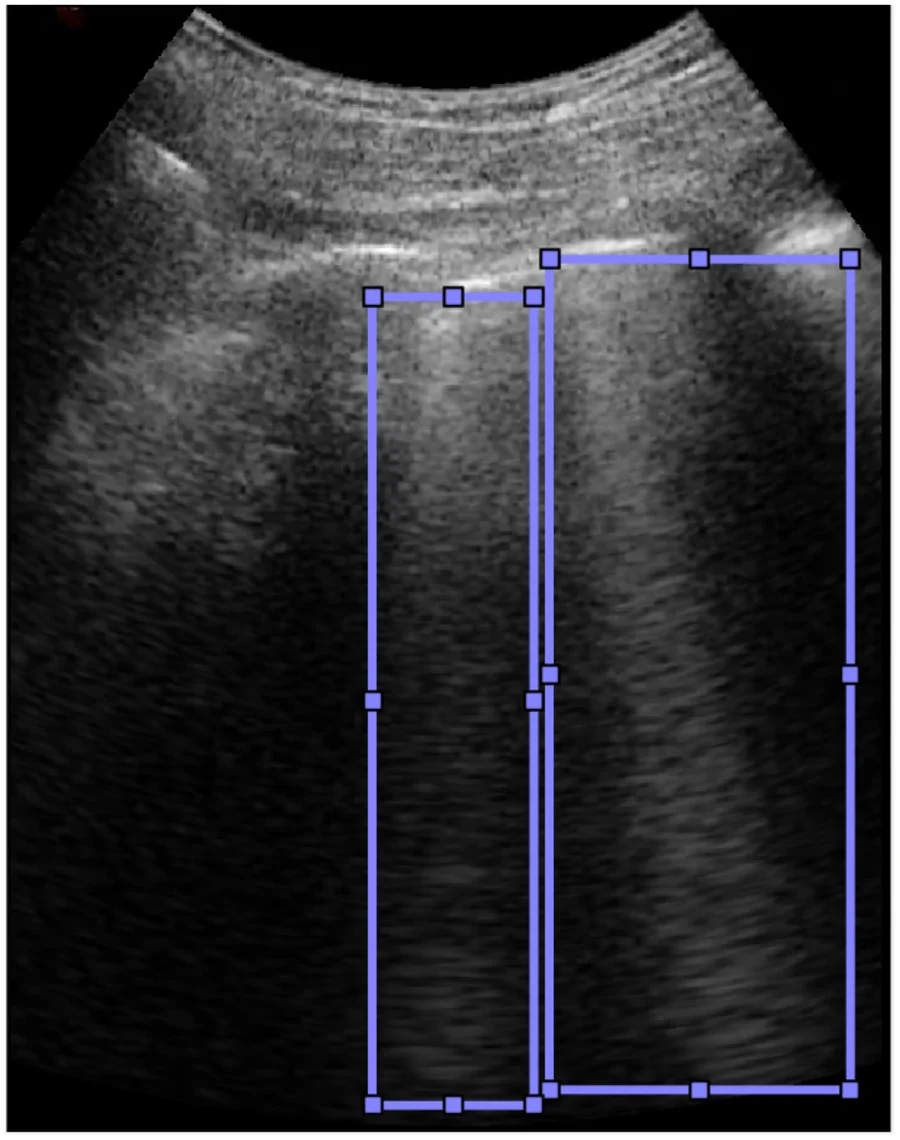
Original LUS image with ground truth annotations represented by purple bounding boxes.

Figure 7 presents a patient's LUS image in which the presence of B-lines must be determined. In this specific case, the image contains two B-lines, indicated by the bounding boxes in Figure 7.

**Figure 8:**
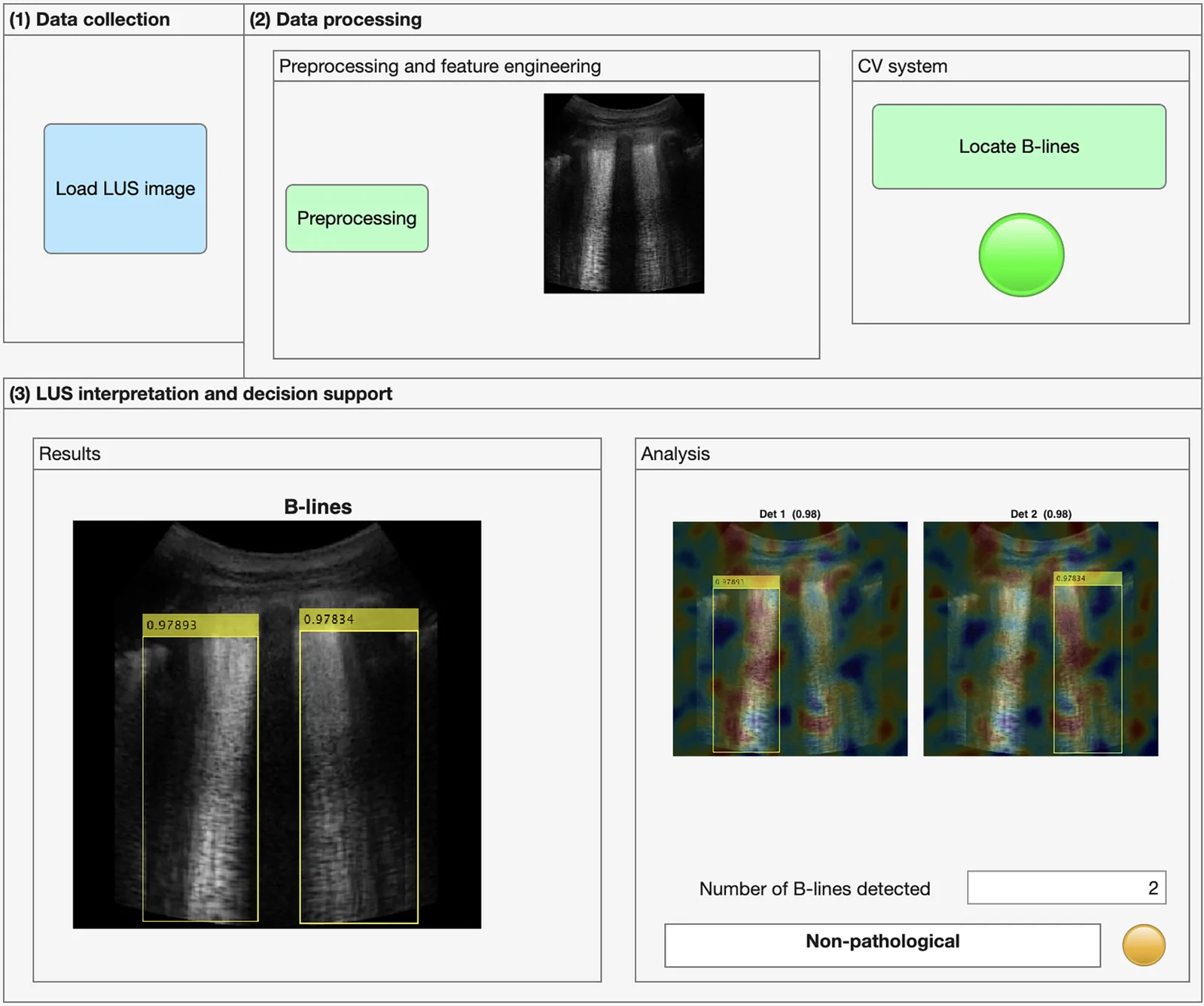
Screenshot of the system interface for the case study.

Figure 8 presents a comprehensive view of the graphical user interface for this practical case.

First, the user loads the LUS image into the application via the “Load image” button in the data collection panel (1). Next, selecting the “Preprocess” button in the processing panel (2) conditions the LUS image for analysis. Finally, the CV system processes the image to identify and localize potential B-lines.

As observed in Figure 8, the model accurately identifies both B-lines with high confidence scores (0.95 and 0.98), as indicated by the two bounding boxes.

In the analysis panel, D-RISE saliency maps (60) are overlaid onto the LUS image. Red regions highlight the areas with the highest influence on the model's predictions, thereby enhancing explainability. In both instances, these highlighted regions directly correspond to the location of the B-lines. Additionally, the lower section displays an automated count of the detected B-lines to support clinical evaluation. For this case, a count of 2 B-lines is reported, which is classified as non-pathological.

## 4 Discussion

LUS is a fundamental diagnostic imaging technique (1) widely used in Critical Care and Emergency Departments (2, 3, 6). Key advantages of LUS include its safety, due to the absence of ionizing radiation, as well as its flexibility and portability, which enable bedside examinations without requiring patient transport (2, 3).

Despite these benefits, the widespread adoption of LUS is hindered by several limitations. Diagnostic interpretation relies primarily on identifying and evaluating US artifacts, such as B-lines, where the presence of three or more B-lines indicates parenchymal pathology (61). However, identifying and quantifying these lines is a labor-intensive, complex process heavily dependent on operator experience and system settings. This inherent subjectivity leads to substantial interobserver variability. Coupled with a shortage of standardized training programs and experts (8, 9), these factors ultimately compromise both the efficiency and reproducibility of the technique.

Despite the widespread availability of US equipment, these limitations have restricted the broader adoption of LUS in areas such as Pulmonology and Primary Care. In this context, AI, particularly CV techniques, offers a promising solution. Although various methodologies have been proposed in the literature, they exhibit substantial heterogeneity (22).

Some studies focus on image segmentation models that perform pixel-level classification of LUS images. While these models offer high precision and are beneficial for applications like 3D biomodel generation or surgical planning (62), their practical utility in LUS is limited as it demands lightweight, efficient systems that enable real-time implementation.

Other studies rely on classification models paired with Grad-CAM saliency maps (63). While this approach highlights the image regions that most influence predictions, thereby aiding artifact localization, it has major limitations. Specifically, classification models are not explicitly designed for structural detection and cannot be quantitatively evaluated using standard detection object metrics such as IoU or AP, hindering objective performance comparisons.

Finally, regarding object detection systems, only four studies were identified (43, 45, 46, 48). These studies exhibit notable limitations, including high variability in detection targets, dataset heterogeneity, and a lack of standardized evaluation metrics. Furthermore, none of these systems were specifically developed for application in Pulmonology or Primary Care.

To address these shortcomings, the present work proposes a CV system based on the YOLO architecture for B-line detection in LUS images. The primary objective is to assist in interpreting LUS findings, facilitate diagnosis and monitoring of respiratory diseases, and promote the broader integration of LUS into clinical practice. While previous works by Bottino et al. (45), Tripathi et al. (46) and Joseph et al. (48) also address B-line detection, their scope is restricted to Emergency and Critical Care settings. Our system advances this approach by not only identifying B-lines but also performing an automated count and classifying each finding as pathological or non-pathological. This functionality simplifies the use of standardized scoring scales and provides an automated assessment of pathological states, defined by the presence of three or more B-lines.

The main contribution of this system lies in the integration of a dedicated preprocessing and feature engineering pipeline, a step absent in existing literature that employs YOLO networks for artifact detection, such as the studies by Bottino et al. (45) and Joseph et al. (48). This pipeline yields an enhanced image that optimizes B-line detection and broadens system versatility. Specifically, the preprocessing stage applies normalization and DGA to homogenize input images, allowing the system to operate effectively across diverse equipment configurations and improving its overall robustness and generalizability. Furthermore, the feature engineering process employs the Radon Transform and its inverse to accentuate key regions of interest, particularly vertical hyperechoic structures, thereby significantly facilitating B-line detection.

Furthermore, the CV system was developed using the LUS-BALD database (50), which comprises 331 LUS images from 152 patients, a sample size considerably larger than those in comparable studies (45, 46, 48). Beyond its size, the LUS-BALD cohort (50) is diverse and representative, encompassing patients with suspected COVID-19 and other pulmonary conditions. This heterogeneity supports the system's clinical utility across a broader diagnostic spectrum. In contrast, Joseph et al. (48) focused exclusively on COVID-19 patients, limiting the generalizability of their system to other respiratory diseases. Similarly, while Tripathi et al. (46) evaluated a heterogeneous cohort with conditions such as hypertension, diabetes, and dyspnea, suitable the Emergency Department or Critical Care, their sample was less specific for fields such as Pulmonology. Lastly, the lack of detailed cohort information in Bottino et al. (45) makes it difficult to objectively assess the scope and applicability of their findings.

Beyond sample representativeness, the technical rigor of data acquisition is equally critical. In this work, LUS images were acquired using a convex probe, which represents the standard modality for pulmonary evaluation. In contrast, studies such as Tripathi et al. (46) and Joseph et al. (48) relied on sector probes, a choice that may constrain the clinical transferability of their findings and the practical adoption of their systems in clinical settings such as Pulmonology.

To optimize system performance, a transfer learning strategy based on the YOLOX architecture (57) was implemented. Specifically, the “Large-COCO” variant was employed, whose CSP-DarkNet-53 (59) backbone is pre-trained on the COCO dataset (58). By avoiding random weight initialization, this approach mitigates the challenges of limited training data and accelerates convergence toward optimal parameters. Furthermore, these architectures are renowned for their speed and ability to handle variable input dimensions. This architectural flexibility not only enhances domain adaptation but also simplifies the training process and their implementation in real-time environments.

Methodologically, the dataset was partitioned into training, validation, and test sets. This inclusion of an independent test set addresses a critical limitation of previous studies, such as Bottino et al. (45), which relied exclusively on training and validation data.

Model performance was evaluated on the test set using standard object detection metrics, including precision, recall, and AP. A key methodological advantage of this work compared to previous studies is the transparent and objective selection of the system's operating point. While existing literature does not specify the confidence threshold (45, 46) or its selection criteria (48), the operating point in this study was rigorously determined by maximizing the F1-score in the test set to achieve an optimal balance between precision and recall. At this optimal confidence threshold of 0.83, the proposed CV system achieved a precision of 89.13%, a recall of 80.39%, and an F1-Score of 84.54%.

These results demonstrate a performance improvement over the two comparable object detection studies (46, 48). Specifically, Tripathi et al. (46) and Joseph et al. (48) obtained precision scores of 67.0% and 83.0%, respectively, with Joseph et al. (48) also reporting a recall of 68.5%. Furthermore, the proposed CV system yielded an AP of 0.82 at an IoU of 0.5. Notably, among the referenced works, only Joseph et al. (48) reported this metric, achieving an AP of 0.65 for B-line detection, further confirming the superior performance of the proposed methodology.

Finally, the integration of D-RISE saliency maps (60) enhances model explainability by highlighting the specific image regions that most influence the system's predictions. When B-lines are identified, these maps localize the artifacts in a manner consistent with clinical literature, thereby supporting the validation of the results.

Overall, the developed CV system serves as an effective, robust tool for automated B-line detection in LUS images, facilitating operator interpretation and mitigating interobserver variability. This capability may support the broader integration of LUS into routine clinical workflows, particularly in settings where it remains underutilized, such as Primary Care and Pulmonology, ultimately optimizing the diagnosis and monitoring of diverse respiratory pathologies.

Furthermore, automating this process reduces the inherent subjectivity of manual artifact analysis, fostering faster and more precise clinical decision-making. From a healthcare management perspective, the system may optimize resource allocation by reducing reliance on costlier or more invasive imaging modalities, such as CT scans or chest radiography.

### 4.1 Limitations

Despite the promising results, several limitations of this study warrant consideration. First, while the LUS-BALD database (50) represents a diverse cohort, its total volume of 331 images remains relatively small. This constrained dataset size limits the application of certain advanced deep learning techniques and elevates the risk of overfitting. Although this shortcoming was mitigated by employing a transfer learning strategy to avoid random weight initialization, using pre-trained networks could constrain the versatility of the final model.

Second, despite being specifically curated for vertical artifact detection across various pathologies, the LUS-BALD database (50) presents inherent contextual restrictions. Beyond its general hospital provenance, the precise clinical settings during data acquisition are unspecified, which could influence model generalizability in specific environments. Furthermore, the prior pre-processing of images by the database creators precluded working directly with raw LUS data.

Third, from a hardware perspective, the system was trained exclusively on data acquired from two commercial US units using convex probes. Given the wide heterogeneity of US equipment and probe types on the market, the system's performance may vary when applied to technical configurations or clinical environments beyond those analyzed in this study.

Fourth, the proposed system functions as a single-class detector focused exclusively on B-lines. Expanding the system's capabilities to perform multi-class detection for a more comprehensive LUS evaluation remains a key objective for future development.

Fifth, the current implementation is restricted to processing static images. However, because the YOLO architecture inherently supports real-time object detection, future investigations should focus on its integration with US hardware to enable live clinical utility.

Sixth, while the evaluation was performed on an independent test set, the limited size of this subset and its origin from the same clinical environment suggest the need for more exhaustive external validations to confirm the robustness of the results. Additionally, due to the very limited number of errors encountered in this test set, a detailed visual analysis of the model's failure cases was beyond the scope of this initial proof-of-concept. Addressing this will be a primary focus in our future studies alongside the creation of a larger proprietary database.

Finally, the inherent lack of transparency in deep learning models based on CNNs remains an ongoing challenge. Although explainability techniques such as D-RISE saliency maps (60) were incorporated, achieving full model interpretability remains a challenge. This ‘black box’ nature currently represents one of the primary barriers to the integration of these systems into daily clinical practice.

## 5 Conclusions

In this work, a CV system specifically designed for the automated detection of B-lines in LUS has been developed. Unlike existing proposals in the literature, this system is conceived for comprehensive application in clinical practice to support the diagnosis and monitoring wide variety of respiratory pathologies, overcoming the limitations of models restricted exclusively to Emergency and Critical Care settings or to specific pathologies such as COVID-19.

As a primary technical contribution, the system innovatively integrates a preprocessing and feature engineering stage aimed at enhancing image quality. This approach provides notable versatility, ensuring the system's compatibility with multiple devices and configuration parameters, thereby reinforcing its robustness in heterogeneous clinical environments. Complementarily, applying a mask based on the Radon Transform and its inverse allows for the precise highlighting of hyperechoic vertical structures, significantly optimizing B-line identification against background noise.

The methodological soundness of this development is supported in the use of the LUS-BALD database (50), which provides a diverse and representative patient cohort, and the implementation of a transfer learning strategy employing the YOLOX ‘Large-COCO’ network. Furthermore, the model was evaluated using standard object detection metrics, specifically precision, recall, IoU and AP, to appropriately address the model's generalization capability.

The results obtained are promising and compare favorably to those reported in the technical literature: for the operating point selected via the F1-score, the system achieves a precision of 89.13%, a recall of 80.39%, and an AP of 0.82 at an IoU of 0.5. These values support the efficacy of the proposal and its potential to facilitate B-line detection in clinical practice. Nevertheless, it should be noted that the system remains in the early stages of development and requires exhaustive validation before clinical integration. Future research will focus on the creation of a proprietary database and the subsequent optimization of the proposed system.

## Data Availability

Publicly available datasets were analyzed in this study. This data can be found here: https://doi.org/10.17632/h923m366sf.2.
